# Analysis of Chlorpyrifos Pesticide Residue in Locally Grown Cauliflower, Cabbage, and Eggplant Using Gas Chromatography–Mass Spectrometry (GC-MS) Technique: A Bangladesh Perspective

**DOI:** 10.3390/foods13111780

**Published:** 2024-06-06

**Authors:** Mysha Momtaz, Mohidus Samad Khan

**Affiliations:** Department of Chemical Engineering, Bangladesh University of Engineering & Technology (BUET), Dhaka 1000, Bangladesh; myshabuet@gmail.com

**Keywords:** pesticide, chlorpyrifos, gas chromatography–mass spectrometry, extraction, validation, maximum residue level, health hazard

## Abstract

Pesticides are chemical substances used to kill or control various types of pests, which are hazardous for crops and animals. Pesticides may remain on or in foods after these are applied to crops. Pesticide residue in food has been a major global concern since there are direct and indirect health hazards associated with the regular consumption of foods with pesticide residues. Chlorpyrifos is one of the most used pesticides that has received much attention worldwide due to its detrimental health impact. The presence of chlorpyrifos residue in food crops can have both long-term and short-term effects on consumer health. Bangladesh is an agricultural country that uses a high volume of pesticides every year including chlorpyrifos. This experimental study aimed to analyze chlorpyrifos pesticide residue in locally grown cauliflower, cabbage, and eggplant samples by gas chromatography–mass spectrometry (GC-MS) technique followed by a suitable extraction process. Commercially available cauliflower, cabbage, and eggplant samples along with samples cultivated with the recommended pesticide dose were collected for qualitative and quantitative analysis. Samples cultivated without chlorpyrifos were collected as control samples for the validation study. The method was validated with respect to accuracy, recovery, reproducibility, linearity, limit of detection, and limit of quantification. The method has a limit of detection (LOD) of 0.011 mg/kg and a limit of quantification (LOQ) of 0.034 mg/kg. The experimental results were compared to the maximum residue level (MRL) to assess the human health impact. Chlorpyrifos residue was found in 44% of cauliflower samples with 91% of samples higher than MRL. The residue was found in 68% of cabbage samples with 53% of samples higher than MRL. For eggplant, the residue was found in 80% of the samples with 65% of samples higher than MRL. The risk assessment based on the residue level found in this study shows a potential health hazard of having a high concentration of chlorpyrifos residue in locally grown vegetables.

## 1. Introduction

Pesticides are an environmental contaminant that has received vast attention due to their wide application in the agricultural sector. Pesticides include insecticides, herbicides, fungicides, rodenticides, miticides, and other growth regulators, which are all chemical compounds applied to crops for field and post-harvest protection to destroy harmful insects, plants, and fungal pests [1,2]. Pesticide residues are often found in food items either from the direct application of pesticides during cultivation or from cross-contamination of other contaminated food items [3]. Pesticides applied in farming contaminate agricultural fields and groundwater, ultimately, reach nearby rivers and water bodies, and damage aquatic ecosystems [4,5]. Overuse of pesticides deteriorates the environment and increases the production cost. Pesticides can pose a significant threat to human health as the crops may retain pesticide residues even after harvest [6]. Furthermore, the health effects of farmers exposed to pesticides are often ignored in developing countries. Often the farmers are not provided with sufficient guidance and instruction on how to protect themselves and the environment from the hazardous effects of pesticides [7]. The past three decades have witnessed serious health and environmental hazards due to the overuse of pesticides. According to the World Health Organization (WHO) and United Nations Environment Program (UNEP), in developing countries, the pesticide poisoning rate is about 2–3 workers per minute and the death of workers is approximately 20,000 per year [4].

Bangladesh is an agricultural country with a total crop production of approximately 27.79 million metric tons [7]. A large part of the population is directly or indirectly related to agriculture. Agriculture employs about 50.28% of the labor force in Bangladesh. Fertilizers, seeds, pesticides, irrigation, and labor are the main agricultural inputs [7]. Agriculture plays as a vital contributor to the economy of Bangladesh. The sector contributes around 19.10% to GDP and the crop sub-sector individually accounts for a 60.83% share of agricultural GDP [8]. Being an agriculture-centric country, the extensive application of pesticides in the commercial cultivation of crops and vegetables in Bangladesh is noteworthy [9]. During the farming season, most farmers spray chemicals and pesticides two days a week. Some farmers even apply pesticides every day and harvest vegetables [7]. Paddy fields alone consume about 70% of pesticides [4]. According to the Bangladesh Agricultural Research Institute (BARI), the applied pesticides are 8–10 times the prescribed amount for vegetables and 10–15 times for fruits [10]. Many pesticides that hold a high share of toxic chemicals, banned by international organizations, are used in Bangladesh [11]. According to a study by the Bangladesh Agricultural Research Institute (BARI), one-third of the pesticides used in Bangladesh are substandard, which strengthens the resistance power of the pests. This is a reason why farmers have to apply excessive doses of pesticides [7]. Lack of knowledge about the recommended withholding period before harvest intensifies this issue. In a survey, it was found that 47% of farmers overdosed on pesticides in crop cultivation, and only 4% of them had formal training on pesticide use and handling. Of the total pesticides used in Bangladesh, organophosphorus compounds comprise 60.4%, carbamates 28.6%, organochlorines 7.6%, and others 3.4%, by chemical composition [12].

Chlorpyrifos, also known as Darsbun, is a broad-spectrum organophosphorus pesticide [13]. It was first introduced and patented in 1965 by Dow Chemical Company. It is used as an insecticide and nematicide [14]. Chlorpyrifos is widely applied to crops against soil insects and arthropods due to its volatility, low solubility in water, and shorter half-life [6,13]. It is registered in Bangladesh for use in crops and vegetables [10,15]. The application rate of chlorpyrifos in Europe is over 50,000 kg/year. In the USA, the rates of agricultural and non-agricultural use of chlorpyrifos are 5 million kg/year and 4 million kg/year, respectively [6]. Chlorpyrifos has been found in food matrices frequently around the world [16,17,18,19,20]. Chlorpyrifos is of moderate toxicity to mammalian species; however, due to the inhibition of the enzyme cholinesterase, it can be extremely hazardous in some cases. Due to its high toxicity, it has been banned by many international organizations [6,21]. Therefore, to ensure food and health safety, assessment of the chlorpyrifos residue in the food matrix is of prime importance. The availability of a rapid, reliable, and inexpensive method is necessary for this purpose.

Various analytical techniques have been developed for the analysis of pesticide residue in food [22]. However, there are limited methods available for both qualitative and quantitative analysis of chlorpyrifos residue in vegetable matrix [22,23]. Gas chromatography–mass spectrometry (GC-MS) is a combined arrangement of chromatographic and spectrometric methods that needs a single instrument to accomplish the whole experiment. The data accuracy using this technique is also higher than other existing techniques.

In this study, qualitative and quantitative analysis of chlorpyrifos residue in cauliflower, cabbage, and eggplant samples collected from agricultural fields and local markets in Bangladesh were performed by a gas chromatography–mass spectrometry method (GC-MS). A suitable extraction process with satisfactory efficiency has also been developed for the extraction of chlorpyrifos from vegetable samples. Further, the method has been validated with respect to key parameters such as accuracy, recovery, repeatability, linearity, limit of detection (LOD), and limit of quantification (LOQ). This study will help officials and food governing bodies in implementing the proper use of chlorpyrifos in agricultural fields.

## 2. Materials and Method

### 2.1. Chemicals and Reagents

The Chlorpyrifos reference standard was purchased from Sigma-Aldrich, St. Louis, MO, USA. The pesticide was provided in a sealed vial. The certified purity of the standard was 98%. HPLC-grade solvent acetonitrile was purchased from Riedel-de Haën Honeywell, Seelze, Germany. The HPLC grade methanol was purchased from VWR Chemicals BDH, Atlanta, GA, USA. Analytical-grade anhydrous sodium sulfate was purchased from Sigma-Aldrich, St. Louis, MO, USA.

### 2.2. Collection and Preservation of Samples

Cauliflowers, cabbages, and eggplants were collected from six different sources. The number of each type of vegetable from each source was five. Each sample was repeated three times during analysis. Control samples were collected from PROSHIKA Agriculture Farm, Tamphat, Rangpur, on 10 February 2021. Field samples were collected from Kashimpur, Gazipur, on 18 March 2021. During this collection, the samples went through all steps of cultivation (3 months) and were ready for transport to the local markets. Samples from local markets of Dhaka were collected on 10 September 2021. The sources are denoted as follows for convenience:

BADC field (Kashimpur): Site—1

Commercial agricultural field (Kashimpur): Site—2

Polashi market (Dhaka): Market—1

Hatirpool market (Dhaka): Market—2

Mohakhali market (Dhaka): Market—3

The sample collection and preservation were performed according to the guidelines for the determination of the levels of pesticide residue for compliance with the maximum residue level (MRL) documented in the EU directive 2002/63/EC [22,24]. 

Composite samples of approximately 500 g each were collected and sealed into individual polybags to avoid cross-contamination. Each sample was labeled with a unique sample code for convenience in further identification. The samples were transported to the Environmental Laboratory of the Chemical Engineering Department, Bangladesh University of Engineering and Technology. The samples were stored in the refrigerator at 4 °C. The whole process was accomplished within 24 h after uprooting the vegetables because the pesticides begin to break down into smaller compounds, and, hence, their analysis might result in inaccuracy. Later, the presence of chlorpyrifos was analyzed in the edible parts of the vegetables.

### 2.3. Extraction of Pesticide

The extraction process was designed by modification of the processes reported in previous studies from other research groups [22,25,26]. Acetonitrile was chosen as the extraction reagent due to its higher solubility than other solvents [27]. Anhydrous sodium sulfate was chosen over anhydrous magnesium sulfate for the removal of excess water because anhydrous magnesium sulfate often agglomerates, which makes it difficult to dissolve [26]. Methanol was used for pigment removal since interference of pigments often intensifies the matrix effect [22].

The dirt was removed by wiping it with dry tissue paper. The samples were whipped with dry tissue paper instead of washed with water to minimize the loss of pesticide residue as much as possible. Approximately 100 g of unwashed fresh samples were chopped by a sterilized knife on the chopping board and then placed into a kitchen hand blender. From the chopped sample, 20 g was weighed into a 100 mL beaker, and 16 mL of acetonitrile was added. The mixture was placed into a shaker at 300 rpm for 6 h. Next, the mixture was transferred into a beaker. The extract was collected by filtration with a nylon membrane of 0.45 micrometer pore size and 5 g of anhydrous sodium sulfate was added. The solution was filtered until the excess water was removed. The filtrate was placed in a separatory funnel and 24 mL methanol was added for pigment removal. The funnel was kept undisturbed for 2 h. The pigmented solution was discarded from the bottom, and the solution from the top was used for further processing.

Further, the solution was transferred to the rotary vacuum evaporator at 250 mbar pressure with a water bath at 45 °C. It was concentrated to 5 mL. The solution was filtered with an Agilent syringe filter of 0.22 mm pore size to ensure the removal of any vegetable matrix if present in the extract. The filtrate was stored in a 50 mL vial in the refrigerator at 4 °C. From the filtrate, 1 mL solution was taken into Agilent certified vial and 1 µL was injected into the gas chromatography–mass spectrometry analyzer.

### 2.4. Gas Chromatography–Mass Spectrometry (GC-MS) Method

The experiment was performed in a gas chromatography (model no. 8890, Agilent Technologies, Mundelein, IL, USA) system coupled with a mass spectrometry (5977B) detector. The machine had a split/splitless injector with an electronic pressure control system. A fused silica DB 5 MS capillary column (30 m × 0.25 mm × 0.25 µm film thickness) was used. Helium was used as carrier gas. Table 1 shows the method in brief.

The MS system was routinely set in selective ion monitoring (SIM) mode. Each compound was quantified based on peak area using three target ions as mentioned earlier. The oven temperature profile for the experiment is given in Table 2.

### 2.5. Preparation of Standards 

Before the injection of the sample extract into GC, a stock solution of 100 mg/L chlorpyrifos was prepared. From the stock solution, standard solutions of 5, 10, 15, and 20 mg/L were prepared into 4 individual vials. The vials were stored in the refrigerator at 4 °C. The standard solutions were injected into the gas chromatography–mass spectrometry analyzer. The oven temperature was selected after observation from several runs with variations in temperature profile. The target peak was characterized by its retention time. The area under the peak versus the concentration was plotted. The data points were fitted using a simple linear regression. Thus, the equation for the standard curve was obtained. The amount of pesticide in each sample was calculated based on the slope of the standard curve.

### 2.6. Pesticide Residue in Field Sample

Concentration of pesticide residue in sample was calculated as,

$$\mathrm{Pesticide}\;\mathrm{in}\;\mathrm{sample}\;(\mathrm{mg}/L)=\frac{Final\;volume\;of\;extract\;\times \;Concentration\;in\;GC-MS}{Volume\;of\;acetonitrile\;added}$$

The pesticide residue was expressed as mg/kg basis, which was compared to the maximum residue level (MRL).

$$\mathrm{Pesticide}\;\mathrm{in}\;\mathrm{sample}\;(\mathrm{mg}/\mathrm{kg})=\frac{Concentration\;of\;pesticide\;in\;sample\;\times \;Volume\;of\;acetonitrile\;added}{Sample\;weight}$$

### 2.7. Validation of Method

The validation study was performed in accordance with the European Commission (EC) guidelines [22]. Vegetable samples cultivated without pesticides were collected from PROSHIKA (Proshika Center for Human Development) organic farm, Rangpur. The samples were confirmed as not containing pesticide residue. The method was validated with respect to accuracy, recovery, reproducibility, linearity, limit of detection (LOD), and limit of quantification (LOQ).

#### 2.7.1. Calibration Curve Properties:

The calibration curve yielded a regression equation of the form, y = mx + c, where m is the slope of the curve and c is the intercept of the y-axis. The regression coefficient (R^2^ value) was evaluated to justify the linear relationship. The curve also had a relative standard deviation, which is a measure of how much the straight line deviated from the origin.

#### 2.7.2. Statistical Analysis 

##### Limit of Detection (LOD) and Limit of Quantification (LOQ)

The limit of detection (LOD) and the limit of quantification (LOQ) [28] are calculated as,

$$\mathrm{Limit}\;\mathrm{of}\;\mathrm{Detection}\;(\mathrm{LOD})=3.3\times \frac{\Sigma }{S}$$

$$\mathrm{Limit}\;\mathrm{of}\;\mathrm{Quantification}\;(\mathrm{LOQ})=10\times \frac{\Sigma }{S}$$

Σ = Standard deviation of the replicates at the lowest point of concentration in the calibration curve

S = Slope of the calibration curve

##### Accuracy

For accuracy study, two vegetable samples were spiked at two concentration levels (6 and 7 mg/L) and the extraction process was performed. The other two samples were spiked at the extraction phase after the extraction process. The extracts were injected into the gas chromatography–mass spectrometry analyzer. 

The accuracy (%) was calculated as,

$$\mathrm{Accuracy}\;(\%)=\frac{Concentration\;in\;sample\;spiked}{Concentration\;in\;extract\;spiked}\times 100$$

##### Recovery and Repeatability

The recovery was studied at 5 different fortification levels (2, 3, 4, 5, and 6 mg/L). The extraction was performed as described in Section 2.3. Each vial was injected four times into the gas chromatography–mass spectrometry analyzer.

The mean recovery (%) was calculated as,

$$\mathrm{Mean}\;\mathrm{Recovery}\;(\%)=\frac{Concentration\;measured}{Concentration\;spiked}\times 100$$

The repeatability (%) was calculated as,

$$\;Repeatability\;(Relative\;Standard\;Deviation)\%\;=\frac{Standard\;deviation\;for\;recovery\;of\;each\;replicate\;set}{Mean\;recovery\;for\;each\;replicate\;set}\times 100$$

## 3. Method Validation Result

### 3.1. Calibration Curve

**Figure 1:**
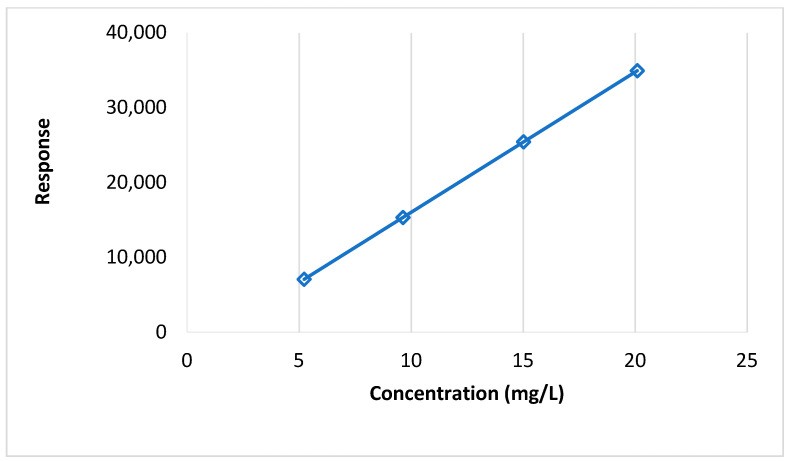
Calibration curve with response and concentration of pesticide.

The calibration curve developed is shown in Figure 1. The curve showed the following characteristics:

Regression equation: y = 1871.9x − 2725.5

Regression coefficient (R^2^ value) = 0.998.

Relative Standard Deviation (RSD) = 4.20%.

### 3.2. Limit of Detection (LOD) and Limit of Quantification (LOQ)

The standard deviation at the lowest point (5 mg/L) was 0.32. The detection limit and quantification limit are shown in Table 3.

### 3.3. Accuracy

The efficiency of the extraction process was determined by an accuracy study. Table 4 presents the concentration in the sample and spiked extract.

**Figure 2:**
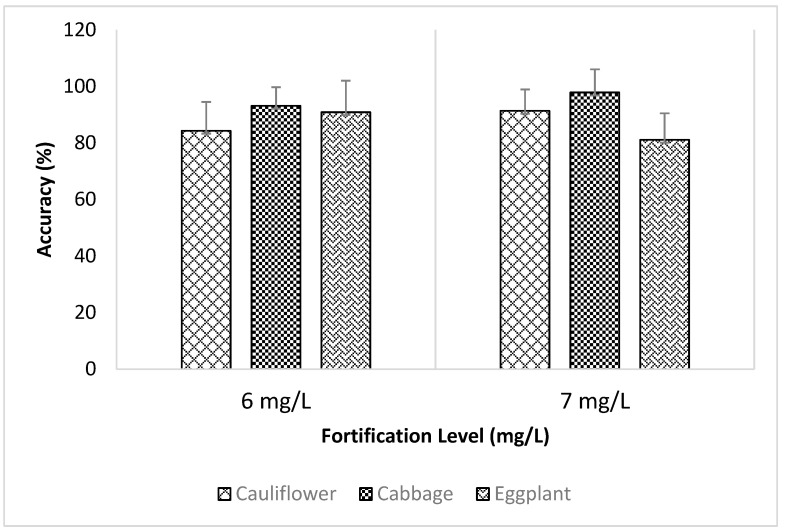
Accuracy study at 6 and 7 mg/L fortification levels.

According to Figure 2, for the 6 mg/L fortification level, eggplant showed the highest accuracy with 90.87% and cauliflower showed the lowest accuracy with 84.30%. For 7 mg/L fortification level, cabbage showed the highest accuracy with 97.85% while eggplant showed the lowest accuracy with 81.14%.

### 3.4. Recovery and Repeatability

The recovery of pesticide shows the matrix effect of the vegetable samples during analysis. According to Table 5, in cauliflower, the highest recovery found for the 2, 3, 4, 5, and 6 mg/L fortification levels was 90.18%, 91.42%, 80.07%, 93.38%, and 89.48%, respectively, while the lowest recovery found was 64.57%, 76.50%, 69.53%, 75.73%, and 83.54%, respectively. In cabbage, the fortification levels mentioned above yielded the highest recoveries at 90.44%, 92.33%, 94.30%, 86.01%, and 92.41%, respectively, while the lowest recovery found was 64.59%, 72.76%, 88.08%, 80.45%, and 73.00%, respectively. In eggplant, for the above fortification levels, the highest recovery found was 95.29%, 85.34%, 91.79%, 99.74%, and 91.44%, respectively, while the lowest recovery found was 68.92%, 80.44%, 77.47%, 85.27%, and 78.43%, respectively.

**Figure 3:**
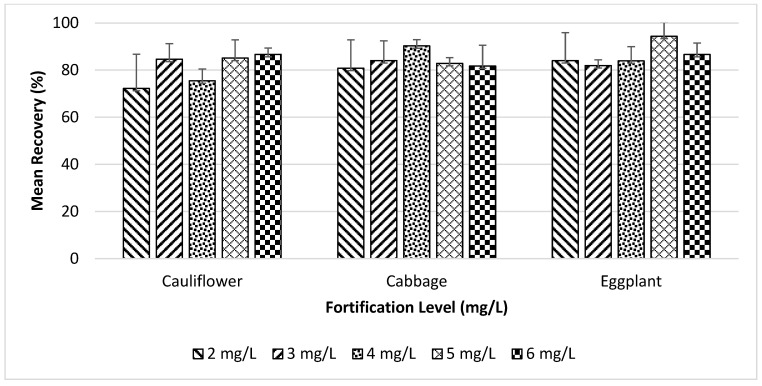
Mean recovery and relative standard deviation study of cauliflower, cabbage, and eggplant samples at 2, 3, 4, 5, and 6 mg/L fortification levels with 4 replicates each.

Figure 3 shows the percentage of mean recovery and relative standard deviation (RSD) for cauliflower, cabbage, and eggplant, respectively. The mean recovery for cauliflower at the 2, 3, 4, 5, and 6 mg/L fortification level was 72.21%, 84.64%, 75.45%, 85.07%, and 86.64%, respectively. The mean recovery for cabbage at the 2, 3, 4, 5, and 6 mg/L fortification level was 80.80%, 84.02%, 90.26%, 82.86%, and 81.74%, respectively. The mean recovery for eggplant at the 2, 3, 4, 5, and 6 mg/L fortification level was 83.98%, 81.93%, 83.89%, 94.34%, and 86.61%, respectively.

## 4. Experimental Results

**Figure 4:**
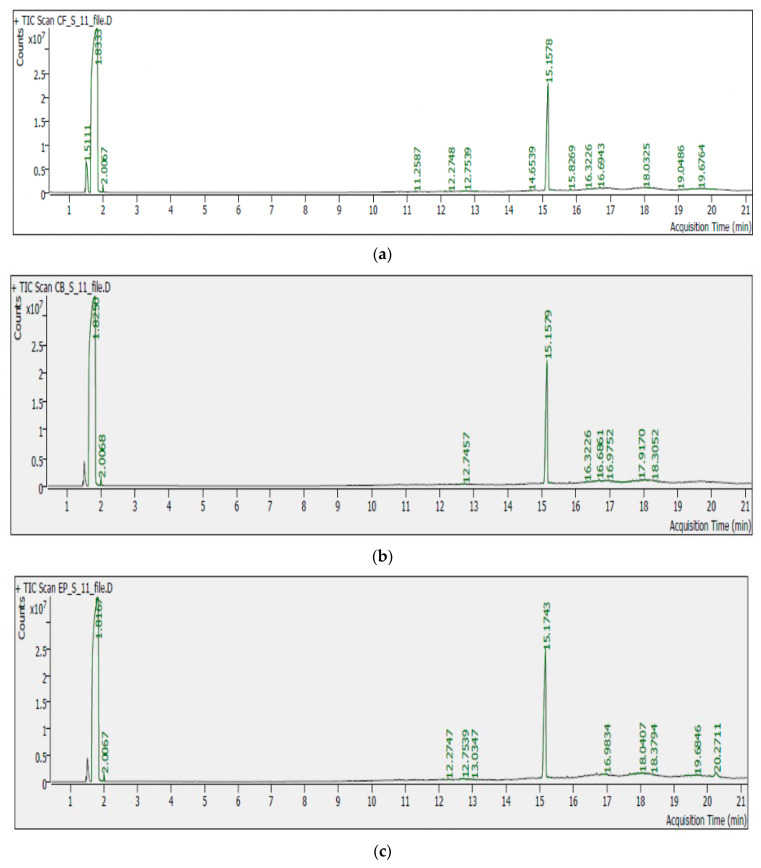
Chromatogram showing (**a**) cauliflower (**b**) cabbage and (**c**) eggplant extract.

The sample extracts produced chromatograms and ion fragmentation patterns. Figure 4 shows the chromatograms of cauliflower, cabbage, and eggplant extract with acquisition time and peak area. 

Chlorpyrifos showed a retention time of 15.16 min. The maximum residue levels (MRL) were considered from EC regulation 396/2005 [29]. It came into effect on 1 September 2008. The maximum residue levels for each type of vegetable are documented in Table 6. Chlorpyrifos residues in samples from sites and local markets are shown in Table 7.

### 4.1. Samples from Site—1

Among the five cauliflower samples collected from BADC farm (site-1), chlorpyrifos was not detected in any of the samples. Among five cabbage samples, chlorpyrifos was detected in three samples and all of those were less than MRL. Among the five eggplant samples collected, chlorpyrifos was detected in two samples with less than MRL as shown in Table 7.

### 4.2. Samples from Site—2

Among the five cauliflower samples collected from the Kashimpur agricultural field (site-2), chlorpyrifos was detected in three samples as shown in Table 7. Out of the three samples, the residue was greater than MRL in two samples. Among the five cabbage samples collected from the Kashimpur agricultural field, chlorpyrifos was detected in all samples, and four of those were greater than MRL. Among the eggplant samples collected, chlorpyrifos was detected in five samples, and the residue was greater than MRL in all of them.

### 4.3. Samples from Local Markets

The cauliflower samples collected from local markets are shown in Table 7. Out of the five samples from market—1, chlorpyrifos was detected in one sample. Out of the five samples from market—2, chlorpyrifos was found in four samples and all of those were greater than MRL. Out of the five samples from market—3, chlorpyrifos was detected in three samples and all of those were greater than MRL. 

Table 7 also shows the cabbage and eggplant samples collected from local markets. Out of the five cabbage samples collected from market—1, chlorpyrifos was detected in three samples, which were less than MRL. Out of the five cabbage samples collected from market—2, chlorpyrifos was found in three samples and all of those were greater than MRL. Out of the five cabbage samples collected from market—3, chlorpyrifos was detected in three samples and two of those were greater than MRL.

Out of the five eggplant samples collected from market—1, chlorpyrifos was detected in five samples and three of those were greater than MRL. Out of the five eggplant samples collected from market—2, chlorpyrifos was detected in four samples and two of those were greater than MRL. Out of the five eggplant samples collected from market—3, chlorpyrifos was detected in four samples and three of those were greater than MRL.

## 5. Discussion

### 5.1. General Discussion

The experimental results showed the residue was greater than the maximum residue level (MRL) in more than half of the positive samples.

**Figure 5:**
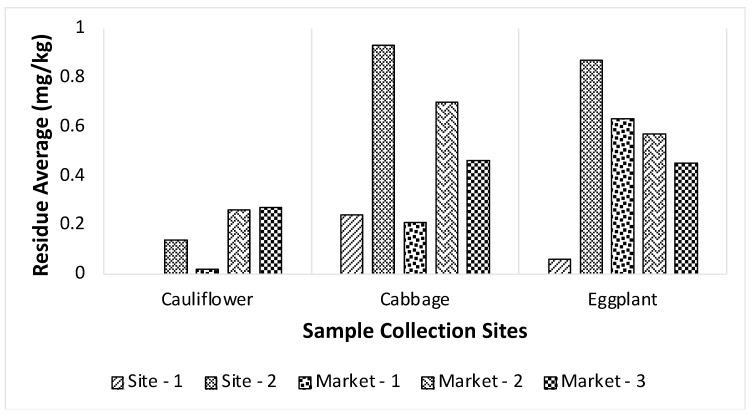
Average residue of chlorpyrifos from collection sites in Cauliflower, Cabbage, and Eggplant.

Figure 5 shows the average pesticide residue in each type of vegetable from each source. Chlorpyrifos residue was found in 33.33% of BADC samples, and chlorpyrifos concentration in all BADC (site-1) samples was less than MRL. Chlorpyrifos residue was found in 86.67% of field (site-2) samples and 84.62% of those samples had chlorpyrifos concentration greater than MRL. Chlorpyrifos residue was also found in 64.44% of samples from local markets, and 55.17% of the positive samples had chlorpyrifos concentration greater than MRL. The chlorpyrifos concentration was the lowest in BADC samples, which indicates the recommended dose and harvest time were followed for BADC samples. Chlorpyrifos residue in cabbage and eggplant samples from the field was found higher than in samples from local markets. Washing or cleaning vegetables by sellers at markets may wash off a portion of pesticide residues from vegetable samples. 

### 5.2. Current Status of Chlorpyrifos Usage in the Target Area

Chlorpyrifos is a widely used pesticide to grow vegetables locally. According to a survey in Kashimpur, many farmers do not follow the recommended dose for chlorpyrifos. According to BADC Kashimpur farm, the average cultivation period of cauliflower, cabbage, and eggplant is three months. Chlorpyrifos is recommended to be applied twice a month. In Kashimpur, farmers spray chlorpyrifos at 3–4 days intervals. The recommended withholding period is two weeks after the pesticide has been applied in the field. However, most farmers do not wait for the recommended withholding period for the harvest to be completed. They usually harvest vegetables 2–3 days after applying the pesticide. Table 8 shows the recommended and applied dosage of chlorpyrifos, which indicates overdosing of chlorpyrifos.

### 5.3. Associated Health Hazards and Risk Assessment

As shown in Table 6, the maximum residue level (MRL) of chlorpyrifos for cauliflower is 0.05 mg/kg. The highest residue level found in the cauliflower samples is 0.27 mg/kg, which is about five times higher than the MRL. The MRL of chlorpyrifos for cabbage is 1 mg/kg. The highest residue level found in the cabbage samples is 0.93 mg/kg, which is close to the MRL. For eggplant, the MRL of chlorpyrifos is 0.5 mg/kg. The highest residue level found in the eggplant samples is 0.87 mg/kg, which is about two times that of MRL (0.5 mg/kg). This study indicates possible health hazards associated with the regular consumption of chlorpyrifos-contaminated vegetables. Chlorpyrifos was found to have severe health impacts. It may enter the animal body by ingestion and contact, and is absorbed through the skin, gut, and pulmonary membranes [6,30]. Even a small amount of chlorpyrifos can cause neurological effects in fetuses and children. An increased risk of delay in mental and motor development at an early age, as well as an increased occurrence of pervasive developmental disorders, is associated with children having been exposed to chlorpyrifos while in the womb. A correlation between parental chlorpyrifos exposure and lower weight and smaller head circumference at birth has also been found [31,32,33]. Another study revealed that people exposed to high levels of chlorpyrifos had autoimmune antibodies that are usually found in people with autoimmune disorders. Therefore, chronic illness related to autoimmune disorders after exposure to chlorpyrifos was proven [34].

Based on the chlorpyrifos residue level identified in this study, the short-term and long-term health risk (Hazard Quotient, HQ) estimations for the dietary intake of vegetables were performed [35,36].

Short-term HQ assessment (aHQ):

The aHQ was calculated from estimated short-term intake (ESTI) and the acute reference dose (ARfD),

$$\begin{array}{c} \mathrm{ESTI}=\frac{the\;highest\;level\;of\;residue\;\times \;food\;consumption}{body\;weight}\\ \mathrm{aHQ}=\frac{ESTI}{ARfD}\times 100\% \end{array}$$

Long-term HQ assessment (cHQ):

The cHQ was calculated from the estimated daily intake (EDI) and the acceptable daily intake (ADI),

$$\begin{array}{c} \mathrm{EDI}=\frac{mean\;level\;of\;residue\;\times \;food\;consumption}{body\;weight}\\ \mathrm{cHQ}=\frac{EDI}{ADI}\times 100\% \end{array}$$

HQ > 1 indicates a potential risk to human health while HQ ≤ 1 shows no risk [37,38,39]. As the individual body weights in population groups, 32 kg body weight for adolescents and 62 kg for adults were chosen [40]. Based on the final report on the Household Income and Expenditure Survey 2016–2017, the food consumption rate of vegetables was 166.1 g capita^−1^ day^−1^ [41]. The aHQ for cauliflower, cabbage, and eggplant was found to be 2.14, 7.30, and 6.82, respectively. The cHQ for these three vegetables was 13.61, 39.96, and 40.59, respectively. The risk assessment shows that HQ was much higher than one for these vegetable samples. 

## 6. Conclusions

Overuse of pesticides is a common phenomenon, especially in developing countries. Therefore, it is important to analyze pesticide residues in foods and associated health hazards. In this experimental study, chlorpyrifos residue was analyzed in cauliflower, cabbage, and eggplant samples by a gas chromatography–mass spectrometry (GC-MS) method followed by a suitable extraction process. The method was validated with respect to accuracy, recovery, reproducibility, linearity, limit of detection, and limit of quantification. Cauliflower, cabbage, and eggplant samples were collected from agricultural fields and local markets. Samples cultivated with minimal pesticide application were collected from BADC Kashimpur farm. Commercially cultivated samples were collected from the agricultural field at Kashimpur and local markets at Polashi, Hatirpool, and Mohakhali. Control samples cultivated without pesticides were collected from the PROSHIKA organic farm. The results obtained through this study indicate the improper use of pesticides in commercially cultivated vegetable samples. From the experimental analysis, it was found that chlorpyrifos concentrations in more than half of the positive samples were higher than the maximum residue level. These results indicate that regular consumption of such vegetables could result in potential health hazards. Regular monitoring of pesticide applications to crops, and developing and implementing appropriate guidelines and regulations in this regard would be useful to improve the situation. This study helps in understanding the status of chlorpyrifos residue in field vegetable samples of Bangladesh. 

## Figures and Tables

**Table 1:** Method for GC-MS analysis of Chlorpyrifos residue in the collected vegetable sample.

Serial	Description	Parameter
1	Injection Mode	Split
2	Injection Ratio	10:1
3	MS Source Temperature	230 °C
4	MS Quadrupole Temperature	150 °C
5	Injector Temperature	250 °C
6	MS Transfer Line Temperature	250 °C
7	MS Heater Temperature	250 °C
8	Ion Source Temperature	250 °C
9	Flow Rate of Carrier Gas	1.2 mL/min at a constant head pressure of 80 pound-force per sq. inch
10	MS Mood	Electron ionization
11	Detector Mood	Mass Selective Detector (MSD)
12	Chromatograms Display	Total Ion Chromatogram (TIC) mode
13	Ion Energy for Electron Impact (EI)	70 eV
14	Presence of Fragment Ions for Identification of Compounds	97, 197, 314

**Table 2:** Oven temperature profile for GC-MS analysis of Chlorpyrifos residue.

Temperature Ramp Rate (°C/min)	Initial Oven Temperature (°C)	Hold Time (min)	Run Time (min)
	49	2	2
67	141	4	7.3731
17	189	4	14.197
15	234	4	21.197

**Table 3:** Limit of detection and limit of quantification.

Detection Limit and Quantification Limit	Chlorpyrifos Residue (mg/kg)
Limit of Detection (LOD)	0.01
Limit of Quantification (LOQ)	0.03

**Table 4:** Concentration of pesticide in the sample and spiked extract.

Sample No.	Fortification Level (mg/L)	Concentration in Sample Spiked (mg/L)	Concentration in Extract Spiked (mg/L)
Cauliflower			
1	6	4.53	5.37
2	7	5.70	6.24
Cabbage			
1	6	4.81	5.16
2	7	6.01	6.14
Eggplant			
1	6	4.95	5.45
2	7	5.01	6.18

**Table 5:** Recovery study of cauliflower at 2, 3, 4, 5, and 6 mg/L fortification levels with 4 replicates each.

	Cauliflower		Cabbage		Eggplant	
Replicate No.	Pesticide Concentration (mg/L)	Recovery (%)	Pesticide Concentration (mg/L)	Recovery (%)	Pesticide Concentration (mg/L)	Recovery (%)
2 mg/L Fortification						
1	1.31	65.31	1.29	64.59	1.63	81.31
2	1.29	64.57	1.81	90.44	1.91	95.29
3	1.38	68.80	1.71	85.35	1.38	68.92
4	1.80	90.18	1.66	82.81	1.81	90.41
3 mg/L Fortification						
1	2.63	87.75	2.77	92.33	2.56	85.35
2	2.49	82.90	2.56	85.37	2.44	81.38
3	2.30	76.50	2.18	72.76	2.42	80.55
4	2.74	91.42	2.57	85.62	2.41	80.44
4 mg/L Fortification						
1	2.78	69.53	3.52	88.08	3.35	83.66
2	3.07	76.64	3.57	89.34	3.67	91.79
3	3.20	80.07	3.57	89.33	3.10	77.47
4	3.02	75.57	3.77	94.30	3.31	82.66
5 mg/L Fortification						
1	4.14	82.70	4.10	82.06	4.72	94.30
2	3.79	75.73	4.02	80.45	4.26	85.27
3	4.67	93.38	4.30	86.01	4.99	99.74
4	4.42	88.47	4.15	82.92	4.90	98.07
6 mg/L Fortification						
1	5.01	83.54	5.55	92.41	5.42	90.24
2	5.37	89.48	4.68	77.92	5.49	91.44
3	5.30	88.32	4.38	73.00	4.71	78.43
4	5.11	85.22	5.02	83.62	5.18	86.32

**Table 6:** Maximum residue level (MRL) of chlorpyrifos for cauliflower, cabbage, and eggplant [29].

Vegetable	Maximum Residue Level (MRL) (mg/kg)
Cauliflower	0.05
Cabbage	1.00
Eggplant	0.50

**Table 7:** Chlorpyrifos residue in samples from sites and local markets.

	Cauliflower		Cabbage		Eggplant	
Sample No.	Chlorpyrifos Residue (mg/kg)	Comment	Chlorpyrifos Residue (mg/kg)	Comment	Chlorpyrifos Residue (mg/kg)	Comment
Site—1						
1	0	----	0.32	<MRL	0.25	<MRL
2	0	----	0	----	0	----
3	0	----	0.53	<MRL	0.05	<MRL
4	0	----	0	----	0	----
5	0	----	0.36	<MRL	0	----
Site—2						
1	0.31	>MRL	1.01	>MRL	1.25	>MRL
2	0	----	1.19	>MRL	0.69	>MRL
3	0	----	0.11	<MRL	1.09	>MRL
4	0.04	<MRL	1.22	>MRL	0.77	>MRL
5	0.34	>MRL	1.11	>MRL	0.53	>MRL
Market—1						
1	0	----	0.47	<MRL	0.67	>MRL
2	0	----	0.05	<MRL	0.26	<MRL
3	0	----	0.51	<MRL	1.19	>MRL
4	0	----	0	----	0.24	<MRL
5	0.11	>MRL	0	----	0.80	>MRL
Market—2						
6	0.10	>MRL	1.17	>MRL	0.11	<MRL
7	0.37	>MRL	0	----	0	----
8	0.28	>MRL	1.05	>MRL	0.66	<MRL
9	0	----	0	----	1.37	>MRL
10	0.56	>MRL	1.28	>MRL	0.71	>MRL
Market—3						
11	0.47	>MRL	0	----	0	----
12	0.36	>MRL	0	----	0.33	>MRL
13	0	----	0.88	<MRL	0.57	>MRL
14	0.52	>MRL	0.24	>MRL	1.04	>MRL
15	0	----	1.20	>MRL	0.31	<MRL

**Table 8:** Recommended and applied chlorpyrifos dosage in Kashimpur area.

Vegetable	Dose Recommended (g/ha)	Dose Applied (g/ha)
Cauliflower	5925	12,345
Cabbage	2962	6419
Eggplant	5925	16,296

## Data Availability

The original contributions presented in the study are included in the article, further inquiries can be directed to the corresponding author.
